# eHealth Literacy Instruments: Systematic Review of Measurement Properties

**DOI:** 10.2196/30644

**Published:** 2021-11-15

**Authors:** Jiyeon Lee, Eun-Hyun Lee, Duckhee Chae

**Affiliations:** 1 College of Nursing Yonsei University Seoul Republic of Korea; 2 Mo-Im Kim Nursing Research Institute Yonsei University Seoul Republic of Korea; 3 Graduate School of Public Health Ajou University Suwon Republic of Korea; 4 College of Nursing Chonnam National University Gwangju Republic of Korea

**Keywords:** eHealth literacy, systematic review, meta-analysis, psychometrics, reliability, validity, scale, instrument

## Abstract

**Background:**

The internet is now a major source of health information. With the growth of internet users, eHealth literacy has emerged as a new concept for digital health care. Therefore, health professionals need to consider the eHealth literacy of consumers when providing care utilizing digital health technologies.

**Objective:**

This study aimed to identify currently available eHealth literacy instruments and evaluate their measurement properties to provide robust evidence to researchers and clinicians who are selecting an eHealth literacy instrument.

**Methods:**

We conducted a systematic review and meta-analysis of self-reported eHealth literacy instruments by applying the updated COSMIN (COnsensus-based Standards for the selection of health Measurement INstruments) methodology.

**Results:**

This study included 7 instruments from 41 articles describing 57 psychometric studies, as identified in 4 databases (PubMed, CINAHL, Embase, and PsycInfo). No eHealth literacy instrument provided evidence for all measurement properties. The eHealth literacy scale (eHEALS) was originally developed with a single-factor structure under the definition of eHealth literacy before the rise of social media and the mobile web. That instrument was evaluated in 18 different languages and 26 countries, involving diverse populations. However, various other factor structures were exhibited: 7 types of two-factor structures, 3 types of three-factor structures, and 1 bifactor structure. The transactional eHealth literacy instrument (TeHLI) was developed to reflect the broader concept of eHealth literacy and was demonstrated to have a sufficient low-quality and very low-quality evidence for content validity (relevance, comprehensiveness, and comprehensibility) and sufficient high-quality evidence for structural validity and internal consistency; however, that instrument has rarely been evaluated.

**Conclusions:**

The eHealth literacy scale was the most frequently investigated instrument. However, it is strongly recommended that the instrument's content be updated to reflect recent advancements in digital health technologies. In addition, the transactional eHealth literacy instrument needs improvements in content validity and further psychometric studies to increase the credibility of its synthesized evidence.

## Introduction

Health literacy is an important determinant for achieving positive health outcomes [1-3]. It refers to the ability to “assess, understand, appraise and apply health information to make judgments and make decisions in everyday life concerning health care, disease prevention and health promotion (p. 3)” [4]. The primary sources for obtaining health information have previously been traditional media (eg, books, brochures, newspapers, and television) and the attending health professionals [5].

The internet is now a major source of health information [6]. There were 5.09 billion internet users worldwide in 2021, representing 64.7% of the global population [7]. In Europe, between 70% and 90% of internet users access health information [8], while about 72% of internet users in the United States search for health information on the internet [9]. Obtaining health information from the internet requires the skills to utilize digital technologies to search and acquire information and basic health literacy abilities such as reading, understanding, and appraising health information. This perspective resulted in the emergence of eHealth literacy in 2006. An early definition proposed for eHealth literacy was “the ability to seek, find, understand, and appraise health information from electronic sources and apply the knowledge gained to addressing or solving a health problem” (p.2) [10].

The rapidly increasing use of digital devices (eg, computers, tablets, and smartphones) and the internet means that health professionals are transiting the method of health information delivery beyond a traditional face-to-face mode into a web-based model, largely due to its advantages of not being restricted to time and space [11]. To ensure the effective web-based delivery of health information, health professionals need to consider the eHealth literacy of consumers. Due to the COVID-19 pandemic requiring quarantining and social isolation, face-to-face visiting of patients with chronic diseases became difficult; therefore, the use of remote care using digital health technologies was recommended as an alternative strategy for delivering health care and informational support [12]. As a result, assessments of eHealth literacy have accelerated as health professionals have attempted to adapt digital health services to patients.

The emergence of eHealth literacy has resulted in the development of self-reporting instruments to measure it. According to the United States Department of Health and Human Services [13], a newly developed or modified self-reporting instrument must satisfy certain measurement properties before applying it in practice or research. Using such an instrument without evidence regarding its measurement properties may misinform practitioners on the measuring concept and threaten the credibility of research results [14]. A systematic review of the measurement properties of eHealth literacy instruments could identify all existing instruments and provide psychometric information to determine which is the best.

One previous narrative review of eHealth literacy instruments [15] simply summarized instruments rather than performing quality assessments or data syntheses. The COSMIN (COnsensus-based Standards for the selection of health Measurement INstruments) is the most popular methodology for systematically reviewing measurement properties of self-reported instruments [16-18]. To the best of our knowledge, such a systematic review of the measurement properties of eHealth literacy instruments has not been conducted previously. Therefore, this study aimed (1) to identify the currently available instruments for measuring eHealth literacy and (2) to evaluate their measurement properties to provide robust evidence for researchers and clinicians to use when selecting instruments.

## Methods

### Design and Searching Strategy

A systematic review of self-reported instruments was conducted according to the updated COSMIN methodology. The PubMed, CINAHL, Embase, and PsycInfo databases were searched from their dates of inception up to March 3, 2021. A search strategy based on the COSMIN involved constructing search filters for the key elements of the construct of interest: population(s), type of instruments (eg, scale or questionnaire), and measurement properties (including inclusion and exclusion filters), and then combining them using AND and NOT Boolean operators. The search filter used for the construct of interest (ie, eHealth literacy) in this study is presented in Multimedia Appendix 1. The search filter for population(s) was not applied because our study aimed to review all self-reported eHealth literacy instruments without considering specific populations. Regarding the type of instruments and the measurement properties, a modified filter developed by the Patient-Reported Outcomes Measurement Group at the University of Oxford and a validated highly sensitive search filter developed using the COSMIN were used [19].

### Eligibility Criteria

We included studies involving the development of an eHealth literacy instrument or evaluations of its measurement properties published as full-text original articles in peer-reviewed journals written in English. If a study had utilized an eHealth literacy instrument as an outcome measure and determined its measurement properties, such as Cronbach’s *α*, but not with the main purpose of evaluating measurement properties of an eHealth literacy instrument, then the article was not included. Literature providing limited information such as conference abstracts, review protocols, or a note were also excluded.

### Selection of Articles

**Figure 1:**
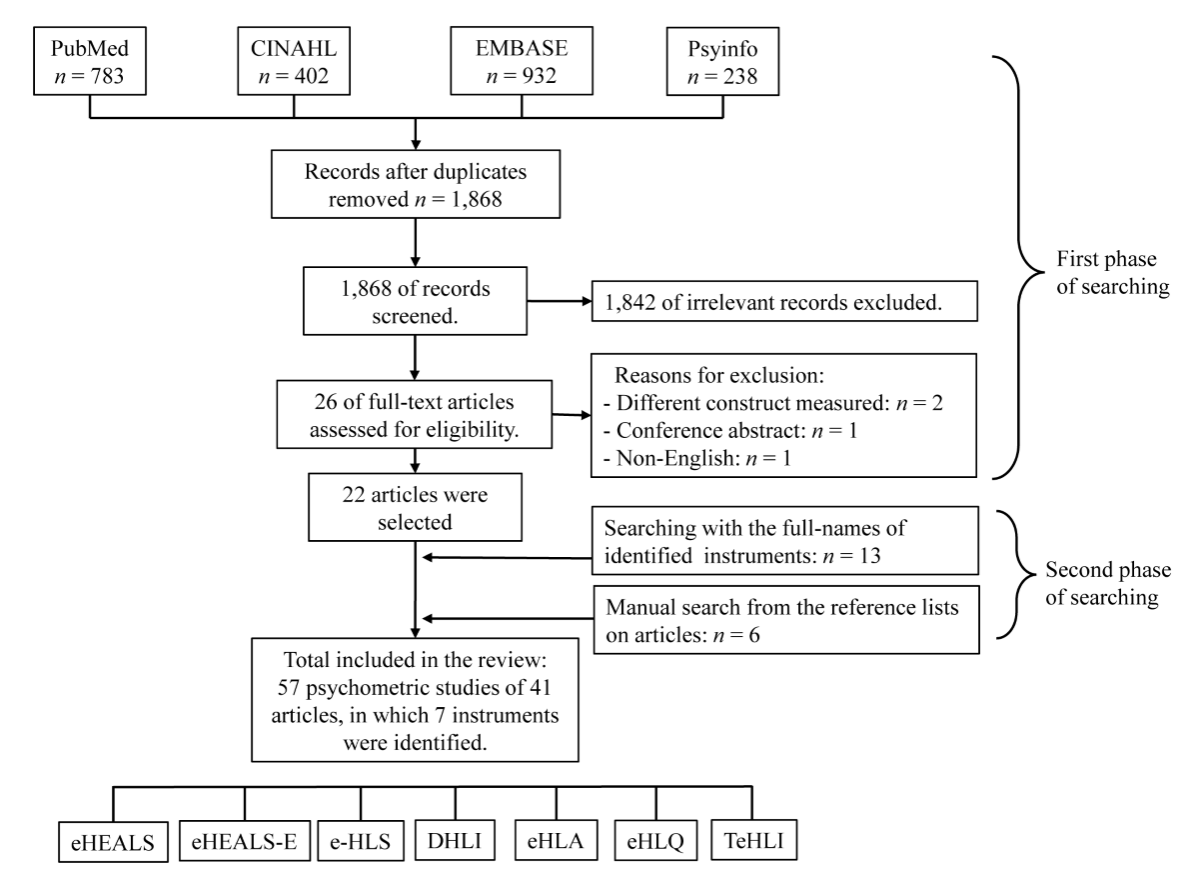
PRISMA flow diagram. DHLI: digital health literacy instrument; eHEALS: eHealth literacy scale; eHEALS-E: eHealth literacy scale–extended; eHLA: eHealth literacy assessment toolkit; e-HLS: electronic health literacy scale; eHLQ: eHealth literacy questionnaire; PRISMA: preferred reporting items for systematic reviews and meta-analyses; TeHLI: transactional eHealth literacy instrument.

Figure 1 presents a flow diagram of PRISMA (preferred reporting items for systematic reviews and meta-analyses) [20]. Duplicated records were removed using EndNote X8.2 (Thomas Reuters). Two reviewers (JL and DC) independently selected articles based on their abstracts and full texts. Differences were discussed, and a consensus was reached by consulting with the third reviewer (E-HL). After identifying an initial list of articles and included instruments in the first phase of searching, database searching utilizing the full names of the identified instruments and the measurement-property filter was conducted in the second phase, which also included manual searching based on the reference lists of the selected articles.

### Data Extraction

Data were extracted from each article to understand the characteristics of the analyzed instrument (ie, target population, number of subscales and items, response options, mode of administration, and language used for the instrument), the study samples (ie, sample size, age, gender) used to assess the identified instruments, theoretical/conceptual frameworks and specified definitions used for the development of instruments, and the results of measurement properties and floor and ceiling effects of the eHealth literacy instruments.

### Evaluating the Measurement Properties of the Instruments

The measurement properties of the instruments were evaluated in 3 steps. First, the methodological quality of the included studies was evaluated using the COSMIN Risk of Bias checklist [16,18]. Each measurement property in each study was evaluated using items in the checklist and rated as very good, adequate, doubtful, or inadequate. The lowest rating of any standard in the box was taken as the methodological quality. Regarding the evaluation of each measurement property, content validity was the first parameter to be evaluated. Content validity (relevance, comprehensiveness, and comprehensibility) was considered the most important measurement property because an instrument needs to reflect the construct being measured adequately. Next, the internal structure of an instrument (structural validity, internal consistency, and cross-cultural validity or measurement invariance) was evaluated. The structural validity of the instrument such as a one-factor or two-factor structure guided the evaluation of internal consistency; for example, when a one-factor structure was supported, then Cronbach’s *α* for all items needed to be evaluated, whereas if a two-factor structure was supported, we needed to evaluate the Cronbach’s *α* of two subscales. Subsequently, remaining measurement properties such as reliability, measurement error, hypotheses testing for construct validity (convergent validity and discriminant or known-groups validity), and responsiveness were evaluated. The methodological quality of criterion validity was not evaluated since there is no gold standard for eHealth literacy measures.

Second, the results of each study for measurement properties were rated according to the updated quality criteria for good measurement properties as sufficient (+), insufficient (–), or indeterminate (?) [18,21]. The quality criteria use only Cronbach’s *α* (≥.70) as the rating indicator of internal consistency. Therefore, the following internal consistency-related criteria were added: (1) sufficient (+) for an omega or person/item reliability of ≥.70 for each unidimensional scale or subscale, insufficient (–) for an omega or person/item reliability of <.70, and indeterminate (?) if the values were not reported; and (2) sufficient (+) for a person/item separation index of ≥1.50 for each unidimensional scale or subscale, insufficient (–) for a person/item separation index of <1.50, and indeterminate (?) if the values were not reported [22]. Additional criteria suggested by Lee et al [23] were applied to evaluate the structural validity obtained in exploratory factor analysis (eg, factor explanation of at least 50% of the variance). The criterion for hypotheses testing (convergent validity) was set as *r*≥.30 with other comparators such as health literacy and internet-related and health-related variables (eg, internet use and adherence to a regimen).

Finally, all of the results for each instrument's measurement properties were qualitatively summarized or quantitatively pooled through meta-analysis using statistical package meta in R software (version 4.0.3; R Core Team). The summarized results related to content validity were rated as sufficient (+), insufficient (–), or inconsistent (±) according to the 10 criteria for good content validity [18]. The summarized or pooled results for other properties were rated as sufficient (+), insufficient (–), inconsistent (±), or indeterminate (?) according to the quality criteria for good measurement properties [17]. Next, the quality of evidence for the overall ratings was graded as high, moderate, low, or very low using the modified GRADE (grading of recommendations assessment, development, and evaluation) approach considering the risk of bias, inconsistency, imprecision, and indirectness [17]. The quality of evidence for structural validity was a prerequisite for analyzing the internal consistency, and so it was taken as a starting point for determining the quality of evidence for internal consistency. The above processes were conducted by all 3 reviewers, with a consensus reached through discussion.

## Results

### Identified eHealth Literacy Instruments

The database search identified 2355 records (783, 402, 932, and 238 in PubMed, CINAHL, Embase, and PsycInfo, respectively), and 1868 records were screened after removing duplicates (Figure 1). In the first phase of searching, 22 articles were selected based on their titles and abstracts. Thirteen articles were identified in the second phase of database searching using the names of the identified instruments and the measurement-property filter, with 6 articles identified through manual searching of the reference lists of the selected articles. Therefore, the total number of included articles was 41. According to the COSMIN, each structure of an instrument is considered a separate study [16]. Some of the identified articles included multiple different factor structures; therefore, 57 studies in 41 articles were finally included in the present systematic review. The following seven instruments were identified: eHealth literacy scale (eHEALS), eHealth literacy scale–extended (eHEALS-E), electronic health literacy scale (e-HLS), digital health literacy instrument (DHLI), eHealth literacy assessment toolkit (eHLA), eHealth literacy questionnaire (eHLQ), and transactional eHealth literacy instrument (TeHLI).

### Characteristics of the Included Instruments and Studies

The characteristics of the included eHealth literacy instruments and studies are presented in Multimedia Appendix 2 [24-64]. The eHEALS, which consists of 8 items scored on a 5-point Likert scale, was originally developed in English [24], and its psychometrics have been studied in diverse languages: Amharic [50], Mandarin Chinese [26], Simplified Chinese [34,36,41], Dutch [25], English [27-30,47,53,54,57,58], German [39,44], Greek [48], Hebrew [45], Hungarian [37], Indonesian [43], Italian [31,33], Korean [32,42,55], Persian [38,46], Polish [35], Portuguese [52], Norwegian [49,56], Serbian [51], and Swedish [40]. The eHEALS has been used to evaluate diverse populations, including not only youths, adults, and older adults, but also healthy people, patients, caregivers, and health professionals in school, community, and clinic settings. The recall period for the eHEALS was specified as “right now,” whereas other instruments did not specify recall periods.

The eHEALS-E is the extended version of the eHEALS comprising 20 items developed for the users of online health communities [59]. The e-HLS, with 19 items, was developed in the United States for online administration to the general population [60]. The DHLI has 21 items scored on a 4-point Likert scale originally developed in Dutch and English and targeting the general population [61]. In addition, this instrument has 3 items that are not obligatory to answer when respondents do not have experience posting messages on social media (ie, they can leave the items blank). The DHLI was further assessed in the Korean language for older adults in welfare centers [42]. The eHLA is the longest instrument, comprising 42 items scored on a 4-point Likert scale and using multiple choices, and was developed in both Danish and English [62]. The eHLQ comprises 35 items scored on a 4-point Likert scale and was also developed in Danish and English [63]. The TeHLI was developed in the United States for patients with lung disease and is composed of 18 items for online administration [64].

### Theoretical/Conceptual Framework, Definition, and Intended Use

The theoretical/conceptual frameworks, definitions used when developing the identified instruments, and intended use are summarized in Table 1. The eHEALS and TeHLI were developed based on the Lily model and self-efficacy theory [10,65] and the transactional model of eHealth literacy (TMeHL) [66], respectively, and their specified definitions of eHealth literacy have been clarified. Both the eHLA and eHLQ were developed based on the eHLF (eHealth literacy framework) [67].

**Table 1:** Theoretical/conceptual framework, specified definition, and intended use.

Instrument	Authors	Theoretical/conceptual framework	Specified definition for the development of the instrument	Intended use
eHEALS^a^	Norman & Skinner [24]	Six components of the Lily model: traditional, computer, information, health, media, and science literacies [10]. Social cognitive theory (self-efficacy theory) [65].	“…the ability to seek, find, understand, and appraise health information from electronic sources and apply the knowledge gained to addressing or solving a health problem (p. 2)” [10].	“…designed to provide a general estimate of consumer eHealth-related skills” (p. 2) [10].
eHEALS-E^b^	Petri et al [59]	—^c^	(Additional items deduced from the definition of the concept used for the eHEALS development were included.)	“…accessing, understanding, appraising, and applying health-related online information” (p. 3) [59].
e-HLS^d^	Seçkin et al [60]	eHealth literacy was grounded on the construct of health literacy, and the three domains of trust, action, and behavior were identified in the literature.	—	“…designed to assess the degree to which people possess the skills required to use eHealth information in an informed way” (p. 3) [60].
DHLI^e^	van der Vaart & Drossaert [61]	The construct of eHealth literacy was derived from formative research of the actual performance tests [68].	—	“…to assess both Health 1.0 and Health 2.0 skills, using self-reporting and performance-based items” (p. 9) [61].
eHLA^f^	Karnoe et al [62]	The constructs of eHealth literacy were from the Lily model as well as the eHLF describing the interaction domains and their relations with individual and system domains [10,67].	—	“…suitable for screening purposes…” (p. 2) [62].
eHLQ^g^	Kayser et al. [63]	Seven-dimension eHLF^h^ [67].	—	“…to support researchers, developers, designers, and governments to develop, implement, and evaluate effective digital health interventions” (p. 7) [63].
TeHLI^i^	Paige et al [64]	TMeHL^j^ [66].	“The ability to locate, understand, exchange, and evaluate health information from online environments in the presence of dynamic contextual factors, and to apply the knowledge gained across ecological levels for the purposes of maintaining or improving health (p. 9).” [66]	“… to measure perceived skills related to the capacity to understand, exchange, evaluate, and apply health information from online multimedia” (p. 738) [64].

^a^eHEALS: eHealth literacy scale.

^b^eHEALS-E: eHealth literacy scale-extended.

^c^Cells left blank if no information was available in the study.

^d^e-HLS: electronic health literacy scale.

^e^DHLI: digital health literacy instrument.

^f^eHLA: eHealth literacy assessment toolkit.

^g^eHLQ: eHealth literacy questionnaire.

^h^eHLF: eHealth literacy framework.

^i^TeHLI: transactional eHealth literacy instrument.

^j^TMeHL: transactional model of eHealth literacy.

### Overall Rating and Quality of Evidence for the Content Validity of Each Instrument

Table 2 presents the overall rating and quality of evidence for content validity for each instrument. The eHEALS was rated as having sufficient moderate-quality evidence for comprehensibility, whereas there was inconsistent low-quality evidence for relevance and insufficient very low-quality evidence for comprehensiveness. The eHEALS-E was rated as having inconsistent moderate-quality evidence for relevance, sufficient very low-quality evidence for comprehensiveness, and inconsistent very low-quality evidence for comprehensibility. The e-HLS, DHLI, eHLA, eHLQ, and TeHLI received sufficient ratings for relevance, comprehensiveness, and comprehensibility with low-quality or very low-quality evidence.

**Table 2:** Overall rating and quality of evidence for the content validity of each instrument.^a^

Instrument	Relevance		Comprehensiveness		Comprehensibility	
	Overall rating	Quality of evidence	Overall rating	Quality of evidence	Overall rating	Quality of evidence
eHEALS^b^	±	Low	−	Very low	+	Moderate
eHEALS-E^c^	±	Moderate	+	Very low	±	Very low
e-HLS^d^	+	Low	+	Low	+	Low
DHLI^e^	+	Low	+	Very low	+	Very low
eHLA^f^	+	Low	+	Low	+	Low
eHLQ^g^	+	Low	+	Low	+	Low
TeHLI^h^	+	Low	+	Very low	+	Low

^a^Sufficient (+), insufficient (−), and inconsistent (±).

^b^eHEALS: eHealth literacy scale.

^c^eHEALS-E: eHealth literacy scale-extended.

^d^e-HLS: electronic health literacy scale.

^e^DHLI: digital health literacy instrument.

^f^eHLA: eHealth literacy assessment toolkit.

^g^eHLQ: eHealth literacy questionnaire.

^h^TeHLI: transactional eHealth literacy instrument.

### Overall Ratings and Quality of Evidence for Other Measurement Properties of Each Instrument

The measurement error and responsiveness were not assessed for any of the instruments; therefore, the results for structural validity, internal consistency, cross-cultural/measurement invariance, reliability, and hypotheses testing (convergent validity and discriminant/known-groups validity) were summarized or pooled for each instrument. The summarized or pooled results for the measurement properties of each instrument are presented in Multimedia Appendix 3. The overall rating and quality of evidence for the properties are presented in Tables 3 and 4.

**Figure 2:**
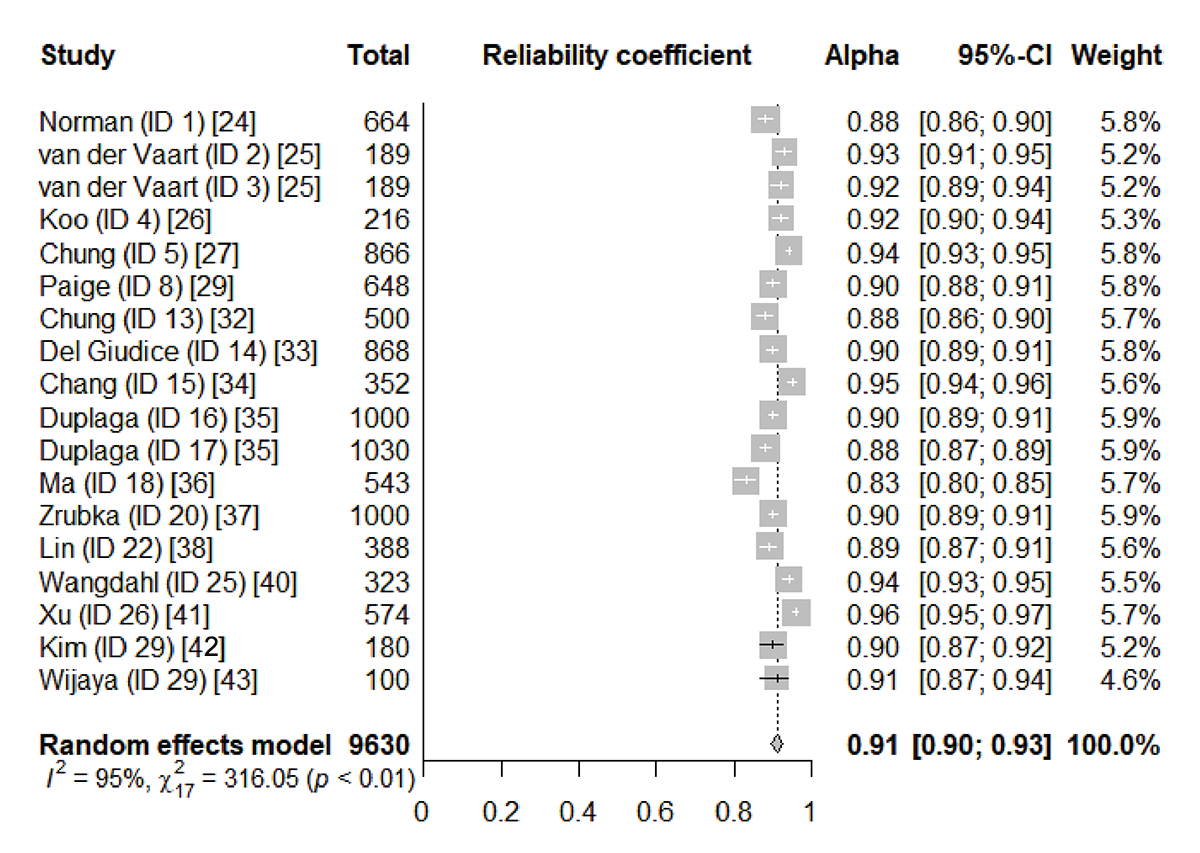
Forest plot of the Cronbach’s alphas for the eight-item single-factor eHEALS. eHEALS: eHealth literacy scale; ID: study identification number.

The single-factor structure of the eHEALS (ID, study identification numbers 1-29) [24-43] demonstrated insufficient moderate-quality evidence (62.1% of the results supported the single-factor structure). Internal consistency of the single-factor eHEALS was supported through a meta-analysis with a Cronbach’s *α* of 0.91 (Figure 2), as well as a qualitative summary with an omega of 0.89-0.94, person reliability of 0.80-0.87, person separation index of 2.36, item reliability index of 0.89-0.93, and item separation index of 3.62-11.3, which were rated as sufficient and indicated that there existed multiple studies of very good quality. According to the COSMIN, the quality of evidence for internal consistency cannot be greater than the quality of evidence for structural validity. Therefore, the quality of evidence for internal consistency was downgraded to moderate to reflect the quality of evidence for structural validity. Measurement invariance for parameters such as gender and age were evaluated in 5 studies and rated as sufficient high-quality evidence. Reliability and hypothesis testing for convergent validity demonstrated that there was insufficient high-quality evidence. There was sufficient moderate-quality evidence for discriminant/known-groups validity.

The second-most-frequent structure of the eHEALS was a two-factor structure. However, the subscale structures were not identical. A two-factor structure as derived from 3 studies (IDs 30-32) [31,39,44] demonstrated insufficient high-quality evidence for structural validity and sufficient high-quality evidence for internal consistency. However, there was inconsistent moderate-quality evidence for convergent validity. The two-factor structure yielded from another 5 studies (IDs 35-39) [47-50] demonstrated insufficient high-quality evidence for structural validity, sufficient high-quality evidence for internal consistency, and sufficient very low-quality evidence for reliability and convergent validity. The three-factor structure of the eHEALS derived from 3 studies (IDs 43-45) [54-56] and a single study ID 47 [58] demonstrated sufficient high-quality evidence for internal consistency, cross-cultural validity, and known-groups validity. The eHEALS derived from 3 studies (IDs 43-45) [54-56] demonstrated insufficient low-quality evidence for reliability and insufficient high-quality evidence for convergent validity, whereas the eHEALS derived from a single study ID 47 [58] did not evaluate these properties; thus no evidence existed.

**Table 3:** Overall rating and quality of evidence for measurement properties of structural validity, internal consistency, and cross-cultural/measurement invariance.^a^

Study ID^b^	Instrument	# of factors	Structural validity		Internal consistency			Cross-cultural/ measurement invariance	
			Overall rating	Quality of evidence	Overall rating	Quality of evidence	Overall rating		Quality of evidence
1-29 [24-43]	eHEALS^c^	1	–	Moderate	+	Moderate	+		High
30-32[31,39,44]	eHEALS	2^d^	–	High	+	High	N/A^e^		N/A
33 [45]	eHEALS	2^f^	+	Low	+	Low	N/A		N/A
34 [46]	eHEALS	2^g^	+	Moderate	N/A	N/A	N/A		N/A
35–39 [47-50]	eHEALS	2^h^	–	High	+	High	N/A		N/A
40 [51]	eHEALS	2^i^	+	Moderate	N/A	N/A	N/A		N/A
41 [52]	eHEALS	2^j^	+	Moderate	+	Moderate	N/A		N/A
42 [53]	eHEALS	2^k^	+	Moderate	+	Moderate	N/A		N/A
43-45 [54-56]	eHEALS	3^l^	+	High	+	High	+		High
46 [57]	eHEALS	3^m^	+	Low	N/A	N/A	N/A		N/A
47 [58]	eHEALS	3^n^	+	High	+	High	+		High
48 [39]	eHEALS	Bifactor^o^	?	Low	N/A	N/A	N/A		N/A
49 [59]	eHEALS-E^p^	6	+	High	+	High	N/A		N/A
50 [60]	e-HLS^q^	3	–	Low	N/A	N/A	N/A		N/A
51 [61]	DHLI^r^	7	+	Low	+	Low	N/A		N/A
52 [42]	DHLI	5	+	Low	N/A	N/A	N/A		N/A
53 [62]	eHLA^s^	7	?	Very low	–	Very low	N/A		N/A
54, 55 [63]	eHLQ^t^	7	–	High	+	High	?		Low
56, 57 [64]	TeHLI^u^	4	+	High	+	High	N/A		N/A

^a^The item numbers of the eHEALS are those assigned in the original article by Norman and Skinner [24].

^b^ID: study identification number (a study identification number was assigned to each of the 57 studies in the 41 articles because some articles covered multiple studies; see Multimedia Appendix 2).

^c^eHEALS: eHealth literacy scale.

^d^Information seeking (items 1, 2, 3, 4, 5, 8), information appraisal (items 6, 7) [31,39,44].

^e^No information was available in the study.

^f^Factor 1 (items 1, 2, 4), factor 2 (items 3, 5, 6, 7, 8) [45].

^g^Factor 1 (items 3, 4), factor 2 (items 1, 2, 5, 6, 7, 8) [46].

^h^Factor 1 (items 1, 2, 3, 4, 5), factor 2 (items 6, 7, 8) [47-50].

^i^Factor 1 (items 2, 6, 7, 8), factor 2 (items 1, 3, 4, 5) [51].

^j^Factor 1 (items 1, 2, 3, 4), factor 2 (items 5, 6, 7, 8) [52].

^k^Information acquisition (items 1, 3, 4), information application (items 2, 5, 6, 7, 8) [53].

^l^Awareness (items 3, 4), skills (items 1, 2, 5), evaluation (items 6, 7, 8) [54-56].

^m^Awareness (items 1, 2), skills (items 4, 5), evaluation (items 6, 7, 8) [57].

^n^Information awareness (items 3, 4), information seeking (items 1, 5), information engagement (items 2, 6, 7, 8) [58].

^o^General factor (items 1, 2, 3, 4, 5, 6, 7, 8), subfactor 1 (items 1, 2, 3, 4, 5, 8), subfactor 2 (items 6, 7) [39].

^p^eHEALS-E: eHealth literacy scale-extended.

^q^e-HLS: electronic health literacy scale.

^r^DHLI: digital health literacy instrument

^s^eHLA: eHealth literacy assessment toolkit.

^t^eHLQ: eHealth literacy questionnaire.

^u^TeHLI: transactional eHealth literacy instrument.

**Table 4:** Overall rating and quality of evidence for measurement properties of reliability, convergent validity, and discriminant/known-groups validity.^a^

Study ID^b^	Instrument	No. of factors	Reliability		Hypothesis testing: convergent validity			Hypothesis testing: discriminant/known-groups validity	
			Overall rating	Quality of evidence	Overall rating	Quality of evidence	Overall rating		Quality of evidence
1-29 [24-43]	eHEALS^c^	1	–	High	–	High	+		Moderate
30-32[31,39,44]	eHEALS	2^d^	N/A^e^	N/A	±	Moderate	N/A		N/A
33 [45]	eHEALS	2^f^	N/A	N/A	+	Moderate	N/A		N/A
34 [46]	eHEALS	2^g^	–	Very low	?	Very low	N/A		N/A
35-39 [47-50]	eHEALS	2^h^	+	Very low	+	Very low	N/A		N/A
40 [51]	eHEALS	2^i^	N/A	N/A	N/A	N/A	N/A		N/A
41 [52]	eHEALS	2^j^	N/A	N/A	N/A	N/A	–		Low
42 [53]	eHEALS	2^k^	N/A	N/A	–	Moderate	N/A		N/A
43-45[54-56]	eHEALS	3^l^	–	Low	–	High	+		High
46 [57]	eHEALS	3^m^	N/A	N/A	N/A	N/A	N/A		N/A
47 [58]	eHEALS	3^n^	N/A	N/A	N/A	N/A	+		High
48 [39]	eHEALS	Bifactor^o^	N/A	N/A	N/A	N/A	N/A		N/A
49 [59]	eHEALS-E^p^	6	N/A	N/A	N/A	N/A	–		Low
50 [60]	e-HLS^q^	3	N/A	N/A	–	Very low	N/A		N/A
51 [61]	DHLI^r^	7	+	Low	–	High	N/A		N/A
52 [42]	DHLI	5	+	Low	–	Low	N/A		N/A
53 [62]	eHLA^s^	7	N/A	N/A	N/A	N/A	N/A		N/A
54, 55 [63]	eHLQ^t^	7	N/A	N/A	N/A	N/A	N/A		N/A
56, 57 [64]	TeHLI^u^	4	N/A	N/A	±	Low	N/A		N/A

^a^The item numbers of the eHEALS are those assigned in the original article by Norman and Skinner [24].

^b^ID: study identification number (a study identification number was assigned to each of the 57 studies in the 41 articles because some articles covered multiple studies; see Multimedia Appendix 2).

^c^eHEALS: eHealth literacy scale.

^d^Information seeking (items 1, 2, 3, 4, 5, 8), information appraisal (items 6, 7) [31,39,44].

^e^No information was available in the study.

^f^Factor 1 (items 1, 2, 4), factor 2 (items 3, 5, 6, 7, 8) [45].

^g^Factor 1 (items 3, 4), factor 2 (items 1, 2, 5, 6, 7, 8) [46].

^h^Factor 1 (items 1, 2, 3, 4, 5), factor 2 (items 6, 7, 8) [47-50].

^i^Factor 1 (items 2, 6, 7, 8), factor 2 (items 1, 3, 4, 5) [51].

^j^ Factor 1 (items 1, 2, 3, 4), factor 2 (items 5, 6, 7, 8) [52].

^k^Information acquisition (items 1, 3, 4), information application (items 2, 5, 6, 7, 8) [53].

^l^Awareness (items 3, 4), skills (items 1, 2, 5), evaluation (items 6, 7, 8) [54-56].

^m^Awareness (items 1, 2), skills (items 4,5), evaluation (items 6, 7, 8) [57].

^n^Information awareness (items 3, 4), information seeking (items 1, 5), information engagement (items 2, 6, 7, 8) [58].

^o^General factor (items 1, 2, 3, 4, 5, 6, 7, 8), subfactor 1 (items 1, 2, 3, 4, 5, 8), subfactor 2 (items 6, 7) [39].

^p^eHEALS-E: eHealth literacy scale-extended.

^q^e-HLS: electronic health literacy scale.

^r^DHLI: digital health literacy instrument.

^s^eHLA: eHealth literacy assessment toolkit.

^t^eHLQ: eHealth literacy questionnaire.

^u^TeHLI: transactional eHealth literacy instrument.

The DHLI, eHLQ, and TeHLI were each psychometrically evaluated twice. Regarding the DHLI, a seven-factor structure (ID 51) [61] yielded sufficient low-quality evidence for structural validity. The high-quality evidence for internal consistency was downgraded to low-quality evidence based on the low-quality evidence for structural validity. There was sufficient low-quality evidence for reliability and insufficient high-quality evidence for convergent validity. The five-factor structure of the DHLI (ID 52) [42] also demonstrated sufficient low-quality evidence for structural validity. The eHLQ from 2 studies reported in a single article (IDs 54, 55) [63] had insufficient high-quality evidence for structural validity, sufficient high-quality evidence for internal consistency, and indeterminate low-quality evidence for measurement invariance. The TeHLI from 2 studies in a single article (IDs 56, 57) [64] demonstrated sufficient high-quality evidence for both structural validity and internal consistency and inconsistent low-quality evidence for convergent validity.

The remaining instruments were assessed only once. The eHEALS-E demonstrated sufficient high-quality evidence for both structural validity and internal consistency and insufficient low-quality evidence for known-groups validity (ID 49) [59]. The e-HLS showed insufficient low-quality evidence for structural validity and insufficient very low-quality evidence for convergent validity (ID 50) [60]. The eHLA exhibited indeterminate, very low-quality evidence for structural validity and insufficient very low-quality evidence for internal consistency (ID 53) [62].

## Discussion

### Principal Findings

This systematic review found that 7 eHealth literacy instruments are currently available. The measurement properties were most frequently assessed for the eHEALS in 18 languages, 26 countries, and diverse populations (eg, patients, adolescents, adults, and the elderly). The conceptualization of a construct to be measured is a basic and initial step when developing a self-reported instrument. The eHEALS was developed based on the definition of “the ability to seek, find, understand, and appraise health information from electronic sources and apply the knowledge gained to addressing or solving a health problem (p. 2)” from the 6 components of literacy in the Lily model: traditional, information, media, health, computer, and science literacies [10]. However, this definition was based on the first generation of simple health information technology (Web 1.0), which later resulted in the eHEALS being criticized as not being sufficiently comprehensive to measure the skills needed for the dynamic and social nature of eHealth (Web 2.0) [68]. One of the researchers who developed the instrument also noticed the lack of social media–related skills being included in the eHEALS, and suggested updating the instrument [69]. In other words, the eHEALS measures eHealth literacy within the restricted scope of the environment before the rise of social media and the mobile web.

Content validity refers to the degree to which the content of an instrument adequately reflects the construct to be measured [70]. The overall ratings of the e-HLS, DHLI, eHLA, eHLQ, and TeHLI for content validity were high but graded as low-quality to very low-quality evidence for relevance, comprehensiveness, and comprehensibility. These findings imply weakness in terms of whether the high ratings are trustworthy and make it difficult to determine which of the instruments has superior content validity.

The eHEALS is a pioneering instrument measuring eHealth literacy and was originally developed with a single-factor structure. However, various other factor structures were identified in this study: 7 types of two-factor structures, 3 types of three-factor structures, and 1 bifactor structure. A possible reason for such diverse factor structures is the instrument contents when considering that insufficient content validity can impair structural validity [18]. The theoretical basis of the eHEALS was the Lily model, which explained multiple components of the constructs. If the contents of the eHEALS effectively reflected the model, the instrument would have been multidimensional. Item variability was also questioned, even though the eHEALS had the same factor structure. These item inconsistencies (Table 3) might be caused by cultural differences that could be closely related to the digital environment of the country in which the study was conducted. The inconsistencies of the factor structures and the corresponding items might also be due to eHEALS already being outdated for use in evaluations at this time, which reflects the dynamic and social nature of eHealth. It was noticed that the eHEALS items do not assess interactive skills when utilizing the internet [61]. Similarly, this systematic review found inconsistent low-quality evidence for relevance and insufficient very low-quality evidence for comprehensiveness in the eHEALS, which might explain its incongruent structures.

The three-factor eHEALS structures reported on in a single study by Paige et al [58] and 3 studies by Sudbury-Riley et al [54], Gartrell et al [55], and Brørs et al [56] were found to be the best structures, with sufficient high-quality evidence for structural validity, internal consistency, measurement invariance, and known-groups validity. Despite these good measurement properties, the three-factor eHEALS structure reported by Sudbury-Riley et al [54], Gartrell et al [55], and Brørs et al [56] demonstrated insufficient low-quality evidence for reliability and insufficient high-quality evidence for hypothesis testing. The three-factor eHEALS by Paige et al [58] has been evaluated only once, so current evidence of its quality is based on the results of the single study (some measurement properties were not evaluated; thus, no evidence existed). Further study is strongly recommended for the suggested three-factor structures of the eHEALS, including reliability, convergent validity, and responsiveness tests. In addition, the three-factor eHEALS has a lack of conceptual comprehensiveness of eHealth literacy. Revising or updating the contents of the eHEALS is therefore recommended to reflect better the skills required for the social nature of eHealth (eg, the sharing of health information).

The eHEALS-E is the extended version of the eHEALS developed to cover better the complex factors contributing to eHealth literacy. However, that instrument was extended under the same definition used for the original version in 2006 [24]. Therefore, this extended version may also be designated as an instrument measuring a narrow scope of eHealth literacy, as for the eHEALS.

Along with the evolution of interactive communication technologies on the internet, conceptual extensions have been demanded for eHealth literacy. This has resulted in the development of second-generation instruments (eg, e-HLS, DHLI, eHLA, eHLQ, and TeHLI) to measure a wider range of eHealth literacy concepts to make them more suitable for people living in the social-media era of eHealth. However, those instruments have been assessed only once or twice, with there being little meaningful synthesized evidence for the measurement properties of each instrument; therefore, further psychometrics studies of them are strongly recommended.

The TeHLI seems to be psychometrically better than the other second-generation instruments. In addition, this is a theory-driven instrument derived from the TMeHL [66] and based on th**e** measurement of transactional features afforded by online media. However, this instrument has only been assessed twice in a single study using classical test theory and item response theory (IRT)/Rasch model with a specific population (ie, baby boomer and older adult patients with chronic lung disease). Therefore, its synthesized evidence for measurement properties cannot be generalized to healthy people or patients of different ages with other diseases. It is therefore suggested that this instrument needs to be assessed in other populations.

### Implications for Future Studies on eHealth Literacy Instruments

The measurement error and responsiveness were not assessed for any of the instruments identified in this study, so future studies of those properties are warranted. More studies of measurement properties also need to be conducted for the second-generation instruments that have been assessed only once or twice. Further psychometric evaluations will increase the credibility of the synthesized evidence. When developing a self-reported instrument, specifying the definition of the concept to be measured is the most basic and important starting point because this determines the scope of the instrument being developed and affects its measurement properties. Nevertheless, the definitions of eHealth literacy were not clarified for most of the instruments identified in this study. New instruments need to be developed for which the definition of eHealth literacy to be measured is clearly addressed, particularly encompassing the attributes/skills required for the social nature of eHealth in the current digital environment.

The assumptions of unidimensionality, local independence, and monotonicity underlying the analyses of structural validity performed using the IRT/Rasch model were not or only partially reported for 11 of 14 studies. According to the COSMIN methodology, the structural validity of a study cannot be rated as sufficient without information about the nonviolation of assumptions underlying IRT/Rasch analysis, even when the model exhibits an adequate fit for structural validity [18,21]. Therefore, clear reports on whether all assumptions are met are needed for future studies that apply IRT/Rasch analysis to assess structural validity.

Convergent validity refers to the relationship of an instrument's score (eg, eHealth literacy instrument) with a comparator instrument that measures similar constructs and has satisfactory measurement properties [71]. The methodological quality of convergent validity was inadequate in 44% of the studies in this review due to no information being provided on the measurement properties of the comparator instrument(s) used for these assessments of eHealth literacy instruments. Future evaluations of convergent validity should therefore employ comparator instruments with satisfactory psychometric properties.

Regarding the instructions provided for how to respond to items, only those for the eHEALS included the recall period: “…tell me which responses best reflect your opinion and experience right now.” Other instruments did not provide information about the recall period, which may result in bias in response items. In the future, it is recommended to provide information about the item response time frame, such as a “short” recall period or the “current state” [72].

### Strengths and Limitations

The first strength of this systematic review is that a two-phase search strategy was performed to exhaustively identify eHealth literacy instruments, as recommended by Lee et al [14], especially when searching for concepts involving compound words such as “eHealth” literacy. The second strength is that internal consistency (Cronbach’s *α*) was qualitatively summarized and quantitatively pooled in a meta-analysis. This is the first meta-analysis applying Cronbach’s *α* to eHealth literacy instruments. A limitation of this study is that it only included peer-reviewed journal articles published in English, which may have resulted in selection bias.

### Conclusions

This systematic review identified 7 eHealth literacy instruments, and complete evaluations of all measurement properties have not been performed for any of these instruments. The eHEALS, based on the 6 components of literacy in the Lily model, was the most frequently investigated instrument with the smallest number of items (8 items), and the 2 three-factor structures of the eHEALS were better than other structures of the instrument; however, this instrument measures a narrow scope of eHealth literacy and so needs to be reconsidered when being applied to people living in the social media era of eHealth (web 2.0). Revising or updating the contents of the eHEALS is necessary to reflect the skills required for the social nature of eHealth. The TeHLI (consisting of 18 items) was the best instrument for broader measurements of eHealth literacy, although it is restricted by generalizing for only healthy people or patients with other diseases in different ages (younger than 40 years). Further psychometric studies of the second-generation eHealth literacy instruments are strongly recommended. In particular, their content validities should be carefully considered due to the results of this systematic review indicating it had low-quality or very low-quality evidence, meaning that they do not fully capture eHealth literacy.

## Appendix

Multimedia Appendix 1 Searching filters.

Multimedia Appendix 2 Characteristics of included instruments and studies.

Multimedia Appendix 3 Summary or pooled results for measurement properties for each instrument.

## Abbreviations

COSMIN: COnsensus-based Standards for the selection of health Measurement INstruments
DHLI: digital health literacy instrument
eHEALS: eHealth literacy scale
eHEALS-E: eHealth literacy scale-extended
eHLA: eHealth literacy assessment toolkit
eHLF: eHealth literacy framework
eHLQ: eHealth literacy questionnaire
e-HLS: electronic health literacy scale
GRADE: grading of recommendations assessment, development, and evaluation
IRT: item response theory
PRISMA: preferred reporting items for systematic reviews and meta-analyses
TeHLI: transactional eHealth literacy instrument
TMeHL: transactional model of eHealth literacy
