# Supplementary material for: eHealth Literacy Instruments: Systematic Review of Measurement Properties
Source: J Med Internet Res. 2021 Nov 15;23(11):e30644. doi: 10.2196/30644 (PMC8663713; doi:10.2196/30644)
Supplement: Multimedia Appendix 1 [file jmir_v23i11e30644_app1.docx]

**Multimedia Appendix 1.** Searching filters.

| ⚫ Construct: eHealth Literacy (compound term of eHealth and health literacy):  - eHealth  ((eHealth[All Fields] OR "electronic health"[All Fields] OR "health technology"[All Fields]) OR ("telecommunications"[MeSH Terms] OR telecommunication [All Fields] OR telecommunications [All Fields] OR teleconference [All Fields] OR telecoaching [All Fields]) OR ("telephone"[MeSH Terms] OR telephone[All Fields]) OR ("educational technology"[MeSH Terms] OR "educational technology"[All Fields]) OR ("communication aids for disabled"[MeSH Terms] OR "communication aids for disabled"[All Fields]) OR ("self-help devices"[MeSH Terms] OR "self-help devices"[All Fields]) OR ("internet"[MeSH Terms] OR internet[All Fields]) OR ("computers, handheld"[MeSH Terms] OR "handheld computers"[All Fields] OR "computers, handheld"[All Fields] OR computers[All Fields]) OR ("electronics"[MeSH Terms] OR electronic[All Fields] OR electronics[All Fields]) OR ("smartphone"[MeSH Terms] OR smartphone[All Fields] OR iPad[All Fields] OR web-based[All Fields] OR ICT[All Fields]) OR ("blogging"[MeSH Terms] OR blogging[All Fields] OR blog[All Fields]) OR ("social media"[MeSH Terms] OR "social media"[All Fields] OR "online social networks"[All Fields] OR "text messaging"[All Fields] OR "social computing"[All Fields] OR "social networking"[All Fields] OR facebook[All Fields] OR twitter[All Fields] OR youtube[All Fields] OR chat[All Fields] OR skype[All Fields]) OR ("communications media"[All Fields] OR multimedia[All Fields] OR e-mail[All Fields] OR "electronic mail"[All Fields] OR "mobile application"[All Fields] OR "mobile device"[All Fields] OR game[All Fields] OR gaming[All Fields] OR "videotape recording"[All Fields] OR video[All Fields] OR technology[All Fields] OR technologies[All Fields] OR telemedicine[All Fields] OR telehealth[All Fields] OR "health information technology"[All Fields] Or digital[All Fields] OR online[All Fields] OR web[All Fields] OR website[All Fields] OR virtual[All Fields]))  - Health Literacy  ("Health Literacy"[MeSH Terms] OR ("communicative literacy" [All Fields] OR "critical literacy" [All Fields] OR "functional literacy" [All Fields] OR "information literacy" [All Fields] OR "health information literacy" [All Fields] OR "numeracy skill" [All Fields] OR "reading skill"[All Fields] OR "health literacy"[All Fields] OR "health literate"[All Fields] OR "health illiteracy"[All Fields] OR "literacy" [All Fields] OR "literate" [All Fields] OR "iliteracy" [All Fields] OR "illiterate" [All Fields])) |
| --- |
| ⚫Type of instruments:  A modified filter developed by the Patient Reported Outcomes Measurement Group, University of Oxford was used (<http://www.cosmin.nl> or <http://phi.uhce.ox.ac.uk/inst_types.php>). |
| ⚫Measurement properties (inclusion and exclusion filters):  A validated highly sensitive search filter developed by Terwee et al. (2009) was used.  Terwee CB, Jansma EP, Riphagen II, de Vet HCW9. Development of a methodological PubMed search filter for finding studies on measurement properties of measurement instruments. Qual Life Res 2009;18:1115–1123. doi: 10.1007/ s11136- 009- 9528- 5. |
