# Supplementary material for: eHealth Literacy Instruments: Systematic Review of Measurement Properties
Source: J Med Internet Res. 2021 Nov 15;23(11):e30644. doi: 10.2196/30644 (PMC8663713; doi:10.2196/30644)
Supplement: Multimedia Appendix 2 [file jmir_v23i11e30644_app2.docx]

**Multimedia Appendix 2.** Characteristics of included instruments and studies.

|  | Instrument | | | | | | | Study | | | | | | | |
| --- | --- | --- | --- | --- | --- | --- | --- | --- | --- | --- | --- | --- | --- | --- | --- |
| ID | Name of instrument | Authors (year) | Target population | (sub)scale(s)  (number of items) | Response options | Recall period | Administration | Sample size | Age (yr)  Mean (SD or range) | % of female | Study population | Setting | Country | Language | O/T |
| 1 | eHEALS | Norman et al. (2006) [24] | General population | Single (8) | 5-point Likert | Right now | Self | 664 | 14.95 (1.24, range=13-21) | 44.3 | Youth | School | Canada | English | O |
| 2 |  | van der Vaart et al. (2011) [25] | Adults | Single (8) | 5-point Likert | Right now | Self | 189 | 52 (11) | 37 | Patients with Rheumatic disease | Community | Netherlands | Dutch | T |
| 3 |  | van der Vaart et al., (2011) [25] | Adults | Single (8) | 5-point Likert | Right now | Self (unclear) | 88 | 43 (18) | 49 | General population | Community | Netherlands | Dutch | T |
| 4 |  | Koo et al. (2012) [26] | Children | Single (8) | 5-point Likert | Right now | Self | 216 | 6th grade of elementary school | 50.2 | Children | School | Taiwan | Chinese (Mandarin) | T |
| 5 |  | Chung et al. (2015) [27] | Older adults | Single (8) | 5-point Likert | Right now | Online | 866 | 62.8 (8.5) | 36.6 | Older adults | Community | USA | English | O |
| 6 |  | Nguyen et al. (2016) [28] | Adults | Single (8) | 5-point Likert | Right now | Self | 164 | (range=18-39) | 79.9 | College students | College | USA | English | O |
| 7 |  | Nguyen et al. (2016) [28] | Adults | Single (8) | 5-point Likert | Right now | Online | 366 | (range >18) | 43.4 | Adults (internet user) | Community | USA | English | O |
| 8, 9 |  | Paige et al. (2017) [29] | Patients with chronic disease | Single (8) | 5-point Likert | Right now | Online | 648 |  | 72.1 | 311 with cardiovascular disease, 131 with arthritis, 181 with mental health disorder, 31 with cancer, 52 with lung disease | Community | USA | English | O |
| 10,11 |  | Stellefson et al. (2017) [30] | Older adults | Single (8) | 5-point Likert | Right now | Telephone | 283 | 67.5 (10.0) | 45.2 | Older adults | Community | USA | English | O |
| 12 |  | Diviani et al. (2017) [31] | General population | Single (8) | 5-point Likert | Right now | Online | 296 | 37.4 (13.8) | 65.2 | Not specified | Community | Switzerland | Italian | T |
| 13 |  | Chung et al. (2018) [32] | Younger adults | Single (8) | 5-point Likert | Right now | Online | 500 | 20-39 | 50 | Young adults | Community | Korea | Korean | T |
| 14 |  | Del Giudice et al. (2018) [33] | Adults | Single (8) | 5-point Likert | Right now | Online | 868 | Group studying or working in the health sector: 31.5 (12.1)  Others: 28.7 (9.7) | 73.4 | Adults | Community | Italy | Italian | T |
| 15 |  | Chang et al. (2018) [34] | Patients with chronic disease | Single (8) | 5-point Likert | Right now | Online | 352 | 29.0 (15.5) | 46.9 | Patients with chronic disease | Clinic | China | Simplified Chinese | T |
| 16,17 |  | Duplaga et al. (2019) [35] | Adults | Single (8) | 5-point Likert | Right now | Telephone and online interview | Sample 1: 1,000  Sample 2: 1,030 | Sample 1: 64.2 (9.6)  Sample 2: 18-35 | Sample 1: 55.8  Sample 2: 100 | Sample 1: at least 50  Sample 2: young adult women (18-35 years) | Community | Poland | Polish | T |
| 18,19 |  | Ma et al. (2019) [36] | Rural people | Single (8) | 5-point Likert | Right now | Interview | 543 | 40.4 (9.2) | 41.4 | Rural adults | Community | China | Simplified Chinese | T |
| 20,21 |  | Zrubka et al. (2019) [37] | Hungarian people | Single (8) | 5-point Likert | Right now | Internet | 1,000 | 46.3 (17.7) | 55.0 | Adults | Community | Hungary | Hungarian | T |
| 22,23 |  | Lin et al. (2020) [38] | Elderly patients with heart failure | Single (8) | 5-point Likert | Right now | Self | 388 | 68.9 (3.4) | 39.7 | Patients with heart failure being aged 65 and over | Clinic | Iran | Persian | T |
| 24 |  | Juvalta et al. (2020) [39] | Parents | Single (8) | 5-point Likert | Right now | Online and Self | 703  (online: 388, paper: 315) | 35.7 (4.3) | - | Parents with children aged 1-24 months | Community | Switzerland | German | T |
| 25 |  | Wångdahl et al. (2020) [40] | Adults | Single (8) | 5-point Likert | Right now | Self | 323 | 49.2 (21.5) | 50 | Adults | Community | Sweden | Swedish | T |
| 26,27 |  | Xu et al. (2020) [41] | Patients | Single (8) | 5-point Likert | Right now | Self | 574 | 45.6 (16.2) | 49.1 | Inpatients | Clinics | China | Simplified Chinese | T |
| 28 |  | Kim et al. (2021) [42] | Older adults | Single (8) | 5-point Likert | Right now | Self | 180 | 73.1 (4.8) |  | Older adults | Welfare centers | South Korea | Korean | T |
| 29 |  | Wijaya et al. (2021) [43] | Adults | Single (8) | 5-point Likert | Right now | Online | 100 | 23.1 (2.8) | 58 | Adults | Community | Indonesia | Indonesian | T |
| 30 |  | Soellner et al. (2014) [44] | Adolescents | Two subscales^a^:  Information-seeking (6),  Information-appraisal (2) | 5-point Likert | Right now | Self | 327 | 18.1 (0.58, range 16-21)  Grade 12th | 64.2 | Students | School | Germany | German | T |
| 31 |  | Diviani et al. (2017) [31] | General population | Two subscales^a^:  Information-seeking (6),  Information-appraisal (2) | 5-point Likert | Right now | Online | 296 | 37.4 (13.8) | 65.2 | Not specified | Community | Switzerland | Italian | T |
| 32 |  | Juvalta et al. (2020) [39] | Parents | Two subscales^a^:  Information seeking (6), information appraisal (2) | 5-point Likert | Right now | Online and Self | 703 (online:388, paper:315 | 35.7 (4.3) | - | Parents with children aged 1-24 months | Community | Switzerland | German | T |
| 33 |  | Neter et al. (2015) [45] | Adults above 21 | Two subscales^b^:  Factor 1 (3)  Factor 2 (5) | 5-point Likert | Right now | Telephone | 199 for EFA  Unknown for CFA | - | - | Adults | Community | Israel | Hebrew | T |
| 34 |  | Bazm et al. (2016) [46] | Youth | Two subscales^c^: Factor 1 (6)  Factor 2 (2) | 5-point Likert | Right now | Self | 525 | - | 69.7 | Students | University | Iran | Persian | T |
| 35 |  | Richtering et al. (2017) [47] | Adults with cardiovascular disease risk | Two subscales^d^:  Knowledge about resource (5)  Evaluation of resource (3) | 5-point Likert | Right now | Self | 397 | 66.3 (8.1) | 23 | Adults with moderate to high cardiovascular disease risk | Clinic | Australia | English | O |
| 36 |  | Efthymiou et al. (2019) [48] | Carers of people with dementia | Two subscales^d^:  Information seeking (5)  Evaluation (3) | 5-point Likert | Right now | Self | 101 | <59: 67.3%, 60-79: 32.7% | 75.2 | Carers of people with dementia | Clinic + community | Greece  Cyprus | Greek | T (modified) |
| 37 |  | Dale et al. (2020) [49] | Patients undergoing day surgery | Two subscales^d^:  Factor 1 (5)  Factor 2 (3) | 5-point Likert | Right now | Self | 109 | <20: 0.9%  20-34: 26.9%  35-49: 25.9%  50-65: 31.5%  >65: 14.8% | - | Patients undergoing day surgery | Clinic | Norway | Norwegian | T |
| 38, 39 |  | Shiferaw et al. (2020) [50] | Patients with chronic disease | Two subscales^d^:  Competency in seeking online health information (5), Appraisal of health information on the internet (3) | 5-point Likert | Right now | Self | 187 | <18 (6.9%), 18-35 (55.10%), >35 (38%) | 36.9 | Patients with chronic disease | Clinic | Ethiopia | Amharic | T |
| 40 |  | Gazibara et al. (2019) [51] | Adolescent | Two subscales^e^:  Factor 1 (4)  Factor 2 (4) | 5-point Likert | Right now | Self | 702 | 16.5 (1.2) | 58.1 | High school students | School | Serbia | Serbian | T |
| 41 |  | Tomas et al. (2014) [52] | Adolescent | Two subscales^f^:  Factor 1 (4)  Factor 1 (4) | 5-point Likert | Right now | Self | 1215 | 16.3 (range=14-22) | 47.1 | Adolescents | School | Portugal | Portuguese | T |
| 42 |  | Holch et al. (2020) [53] | University students | Two subscales^g^:  Information acquisition (3), information application (5) | 5-point Likert | Right now | Self | 188 | 20.1 (2.2) | 88.8 | University students | University | UK | English | O |
| 43 |  | Sudbury-Riley et al. (2017) [54] | Baby boomers (1946-1964) | Three subscales^h^: Awareness (2), Skills (3), Evaluation (3) | 5-point Likert | Right now | Online assumed | UK: 407  New Zealand: 276  USA: 313 | UK: 59.6 (5.2)  New Zealand: 61.3 (5.8)  USA: 60.3 (5.4) | UK: 52.8  New Zealand: 48.9  USA: 47.9 | Baby boomers (1946-1964) | Community | UK, New Zealand, USA | English | O |
| 44 |  | Gartrell et al. (2020) [55] | Nurses | Three subscales^h^: Awareness (2), Skills (3), Evaluation (3) | 5-point Likert | Right now | Self | 484 | 30.8 (6.6) | 98.6 | Hospital nurses | Clinic | South Korea | Korean | T |
| 45 |  | Brørs et al. (2020) [56] | Patients undergone percutaneous coronary intervention | Three subscales^h^: Awareness (2), Skills (3), Evaluation (3) | 5-point Likert | Right now | Self | 1,695 | 66 (10) | 22.54 | Patients undergone percutaneous coronary intervention | Clinic | Norway | Norwegian | T |
| 46 |  | Hyde et al. (2018) [57] | MRI and CT medical imaging outpatients | Three subscales^h^: Awareness (2), Skills (3), Evaluation (3) | 5-point Likert | Right now | Online | 268 | 53 (15) | 55.2 | MRI and CT medical imaging outpatients | Clinic | Australia | English | O |
| 47 |  | Paige  Et al. (2018) [58] | Adults | Three subscales^i^:  Information awareness (2), Information seeking (2), Information engagement (4) | 5-point Likert | Right now | Online | 829  (Millennials: 281, Generation X: 164, Baby boomers/silent generation: 384) | Millennials: 26.7 (5.1), Generation X: 43.0 (5.0) Baby boomers/silent generation: 62.8 (6.7) | 72.7  (Millennials: 73.7 Generation X: 67.1,  Baby boomers/silent generation: 74.3) | Adults | Community | USA | English | O |
| 48 |  | Juvalta et al. (2020) [39] | Parents | Bifactor^j^:  general (8), subfactor 1 (6), subfactor 2 (2) | 5-point Likert | Right now | Online and Self | 703  (online: 388, paper: 315) | 35.7 (4.3) | - | Parents with children aged 1-24 months | Community | Switzerland | German | T |
| 49 | eHEALS-E | Petri et al. (2017) [59] | User of online health communities | Six subscales:  Awareness of sources (3), Recognizing quality and meaning (3), Understanding information (4), Perceived efficacy (4), Validating information (3), being smart on the Net (3) | 5-point Likert |  | Online | 644 | 40 (10.3) | 83.0 | User of online health communities | Community | Slovenia | Unclear | Unclear |
| 50 | e-HLS | Seꞔkin et al. (2016) [60] | Adults | Three subscales:  Action (13), Trust (4),  Communication (2) | 5-point Likert |  | Online | 710 | 48.8 (16.4) | 53.7 | General population | Community | USA | English | O |
| 51 | DHLI | van der Vaart et al. (2017) [61] | General population | Seven subscales: Operational skills (3), Information searching (3), Evaluating reliability (3), Determining relevance (3), Navigation skills (3), Adding content (3), Protecting privacy (3, not obligatory to fill in) | 4-point Likert:  from “very easy” to “very difficult”/ from “never” to “often” |  | Self | 200 | 46.4 (19.0) | 53.5 | General Dutch population | Community | Netherland | Dutch (English version) | O |
| 52 |  | Kim et al. (2021) [42] | Older adults | Five subscales:  Operation skills (3), information searching (9), navigation skills (3), adding content (3), protecting privacy (3) | 4-point Likert:  from “very easy” to “very difficult”/ from “never” to “often” |  | Self | 180 | \| 73.1 (4.7) \|  \| Older adults \| Welfare centers \| South Korea \| Korean \| T \| \| --- \| --- \| --- \| --- \| --- \| --- \| --- \| |  | Older adults | Welfare centers | South Korea | Korean | T |
| 53 | eHLA | Karnoe et al. (2018) [62] | Adults | Seven subscales  Functional health literacy (10), Self-assessed health literacy (9), Familiarity with health and health care (5), Knowledge of health and disease (4), Familiarity with technology (6), Technology confidence (4), Incentives for engaging with technology (4) | 4-point Likert and multiple choice (correct/incorrect/don’t know) |  | Self and online | 475 | 18-35: 30.9%, 36-60: 36.6%,  60+: 28.0% | 51.6 | Healthy people and patients | Community and clinic | Denmark, | Danish, (English version) | O |
| 54,55 | eHLQ | Kayser et al. (2018) [63] | Adults | Seven subscales:  Using technology to process health information (5), Understanding of health concepts and language (5), Ability to actively engage with digital services (5), Feel safe and in control (5), Motivated to engage with digital services (5), Access to digital services that work (6), Digital services that suit individual needs (4) | 4-point Likert |  | Self and online | 475 |  |  | Healthy people and patients | Community and clinics | Denmark | Danish and English | O |
| 56,57 | TeHLI | Paige et al. (2019) [64] | Patients with lung disease | Four subscales:  Functional (4), Communicative (5), Critical (5), Transactional (4) | 5-point Likert |  | Online | 283 | 64.3 (10.5) | 42.4 | Patients with chronic lung disease who are baby boomers and older adults | Community | USA | English | O |

ID, study identification number (a study identification number was assigned to each of the 57 studies in the 41 articles because some articles covered multiple studies); Administration (self-reported paper- and pencil, online, telephone, and interview); O/T, Original or translated version; SD, standard deviation; EFA, exploratory factor analysis; CFA, confirmatory factor analysis.

DHLI, Digital Health Literacy Instrument; eHEALS, eHealth Literacy Scale; eHEALS-E, eHealth Literacy Scale-Extended; eHLA, eHealth Literacy Assessment Toolkit; eHLQ, eHealth Literacy Questionnaire; e-HLS, electronic Health Literacy Scale; TeHLI, Transactional eHealth Literacy Instrument.

(continued)

|  | Instrument |  | Study (continued) |  |  |
| --- | --- | --- | --- | --- | --- |
| ID | Name of instrument | Authors (year) | Mean (SD) | Floor effect | Ceiling effect |
| 1 | eHEALS | Norman et al. (2006) [24] |  |  |  |
| 2 |  | van der Vaart et al. (2011) [25] | 28.2 (5.9) | Reported as “acceptable” | Reported as “acceptable” |
| 3 |  | van der Vaart et al., (2011) [25] | 27.6 (5.9) |  |  |
| 4 |  | Koo et al. (2012) [26] | 28.4 (7.6) | 6.5% | 3.2% |
| 5 |  | Chung et al. (2015) [27] | 30.94 (6.0) |  |  |
| 6 |  | Nguyen et al. (2016) [28] |  |  |  |
| 7 |  | Nguyen et al. (2016) [28] |  |  |  |
| 8, 9 |  | Paige et al. (2017) [29] | 30.34 (5.3) |  |  |
| 10,11 |  | Stellefson et al. (2017) [30] | 29.05 (5.8) |  |  |
| 12 |  | Diviani et al. (2017) [31] | 26.65 (6.3) |  |  |
| 13 |  | Chung et al. (2018) [32] | 28.06 (4.8) |  |  |
| 14 |  | Del Giudice et al. (2018) [33] | 31.9 (5.9), 26.7 (5.6) |  |  |
| 15 |  | Chang et al. (2018) [34] |  |  |  |
| 16,17 |  | Duplaga et al. (2019) [35] | 25.26 (5.9), 29.46 (5.1) |  |  |
| 18,19 |  | Ma et al. (2019) [36] |  |  |  |
| 20,21 |  | Zrubka et al. (2019) [37] | 29.16 (5.2) |  |  |
| 22,23 |  | Lin et al. (2020) [38] |  | 1.0% | 2.8% |
| 24 |  | Juvalta et al. (2020) [39] | 28.5 (6.2) |  |  |
| 25 |  | Wångdahl et al. (2020) [40] | 29.3 (6.2) |  |  |
| 26,27 |  | Xu et al. (2020) [41] | 29.26 (6.8) | 1.8% | 10.6% |
| 28 |  | Kim et al. (2021) [42] | 3.88 (0.5)^a^ |  |  |
| 29 |  | Wijaya et al. (2021) [43] | 30.46 (5.1) |  |  |
| 30 |  | Soellner et al. (2014) [44] |  |  |  |
| 31 |  | Diviani et al. (2017) [31] | 26.65 (6.3) |  |  |
| 32 |  | Juvalta et al. (2020) [39] | 28.5 (6.2) |  |  |
| 33 |  | Neter et al. (2015) [45] |  |  |  |
| 34 |  | Bazm et al. (2016) [46] |  |  |  |
| 35 |  | Richtering et al. (2017) [47] | 27.1 (6.7) |  |  |
| 36 |  | Efthymiou et al. (2019) [48] | 29.27 (5.3) |  |  |
| 37 |  | Dale et al. (2020) [49] | 29.0 (5.1) |  |  |
| 38, 39 |  | Shiferaw et al. (2020) [50] |  | Reported as “acceptable” | Reported as “acceptable” |
| 40 |  | Gazibara et al. (2019) [51] |  |  |  |
| 41 |  | Tomas et al. (2014) [52] | 3.46 (0.6)^a^ |  |  |
| 42 |  | Holch et al. (2020) [53] | 29.46 (4.9) | 16.48% | 28.12% |
| 43 |  | Sudbury-Riley et al. (2017) [54] |  |  |  |
| 44 |  | Gartrell et al. (2020) [55] |  |  |  |
| 45 |  | Brørs et al. (2020) [56] | 25.66 (6.2) | 2% | 3% |
| 46 |  | Hyde et al. (2018) [57] |  |  |  |
| 47 |  | Paige et al. (2018) [58] |  |  |  |
| 48 |  | Juvalta et al. (2020) [39] | 28.5 (6.2) |  |  |
| 49 | eHEALS-E | Petri et al. (2017) [59] |  |  |  |
| 50 | e-HLS | Seꞔkin et al. (2016) [60] | 2.51 (0.8) |  |  |
| 51 | DHLI | van der Vaart et al. (2017) [61] | 3.11 (0.5) |  |  |
| 52 |  | Kim et al. (2021) [42] | 2.86 (0.5) |  |  |
| 53 | eHLA | Karnoe et al. (2018) [62] |  |  |  |
| 54,55 | eHLQ | Kayser et al. (2018) [63] |  |  |  |
| 56,57 | TeHLI | Paige et al. (2019) [64] |  |  |  |

ID, study identification number (a study identification number was assigned to each of the 57 studies in the 41 articles because some articles covered multiple studies); Administration (self-reported paper- and pencil, online, telephone, and interview); O/T, Original or translated version; SD, standard deviation; EFA, exploratory factor analysis; CFA, confirmatory factor analysis.

DHLI, Digital Health Literacy Instrument; eHEALS, eHealth Literacy Scale; eHEALS-E, eHealth Literacy Scale-Extended; eHLA, eHealth Literacy Assessment Toolkit; eHLQ, eHealth Literacy Questionnaire; e-HLS, electronic Health Literacy Scale; TeHLI, Transactional eHealth Literacy Instrument.

^a^, The average score of each eHEALS item was calculated instead of a sum score
