# Supplementary material for: eHealth Literacy Instruments: Systematic Review of Measurement Properties
Source: J Med Internet Res. 2021 Nov 15;23(11):e30644. doi: 10.2196/30644 (PMC8663713; doi:10.2196/30644)
Supplement: Multimedia Appendix 3 [file jmir_v23i11e30644_app3.docx]

**Multimedia Appendix 3.** Summary or pooled results for measurement properties for each instrument.

| Instrument (study identification number) | Structural validity | Internal consistency | Cross-cultural/ measurement invariance | Reliability | Hypotheses testing: convergent validity | Hypotheses testing: discriminant, known-groups validity |
| --- | --- | --- | --- | --- | --- | --- |
| eHEALS  (ID 1-29) [24-43] | 1 factor: (*n*=15,552)  18 out of 29 were confirmed using EFA, CFA, and IRT/Rasch analysis. | (Total: *n*≒11,624)  <Quantitative pooling> Cronbach’s alpha: 0.91 (95% CI: .90-.93 (*n*=9,630).  <Qualitative summary>  Omega: .89-.94 (*n*=1,925)  Person reliability: .80-.87 (*n*=1,214)  Item reliability: .89-.93 (*n*=1,319)  Person separation index: 2.36 (*n*=388)  Item separation index: 3.62-11.3 (*n*=1,319) | 4 out of 5 studies were confirmed  (*n*≒2,349) | Total: (*n*≒2,104)  <Pearson correlation>  *r*=.75-.85 over 75 minutes to 1 month (*n*≒1.064)  <ICC>  ICC=.49-.77 over 75 minutes to 1 month (*n*≒803).  <Kappa>  Weighted quadratic kappa (items): .64-.79 (*n*=35) | 27 out of 38 hypotheses were confirmed (*n*=6,290):  -with age  -with computer/internet related comparators (internet use, actual performance, computer skills, web use, online health information-seeking behaviors, use of digital media, amount of time spent online etc.)  -with health-related comparators (perceived health, mental health, adherence to regimen etc.)  -with health literacy | 10 out of 10 hypotheses were confirmed (*n*=3,104).  -with internet use on PC (one vs. more hours)  -with internet use on phone (one vs. more hours)  -Groups by readiness to use the internet for researching health-related information  -with Groups by readiness to use the internet as a primary source of information  -with 6 items of the health-related activities (5 item were significantly different)  -with age groups |
| eHEALS  (ID 30-32)  [44, 31, 39] | 2 factors^a^: (*n*=1,326)  2 out of 3 were confirmed using CFA | Cronbach’s alpha: (*n*=327)  Information seeking: .88  Information appraisal: .83 |  |  | 12 out of 23 hypotheses were confirmed (*n*=444):  -with age  -with computer/internet related comparators (online behaviors, health information seeking on the Web, use of internet, seeking health information by surfing the net etc.)  -with health-related comparators (health competency, self-efficacy, eHealth predisposition etc.) |  |
| eHEALS  (ID 33) [45] | 2 factors^b^: (n≒199)  1 out of 1 was confirmed using CFA | Cronbach’s alpha:  (*n*≒199)  Factor 1: .83, Factor 2: .83 |  |  | 1 out of 1 hypothesis was confirmed (*n*≒199):  -with health literacy *r*=.36 |  |
| eHEALS  (ID 34) [46] | 2 factors^c^: (*n*=525)  1 out of 1 was confirmed using EFA |  |  | ICC=.56 (*n*=30) | 1 out of 1 hypothesis was indeterminate (*n*=525):  -with computer literacy |  |
| eHEALS  (ID 35-39)  [47-50] | 2 factors^d^: (*n*=981)  2 out of 5 were confirmed using EFA, 1 CFA, ad IRT | Cronbach’s alpha: (*n*=288)  Factor 1: .77-.95, Factor 2: .78-.83 |  | ICC=.92 (*n*=74) | 1 out of 1 hypothesis was confirmed (*n*=187):  -with computer literacy: *r*=.68 |  |
| eHEALS  (ID 40) [51] | 2 factors^e^: (*n*=702)  1 out of 1 was confirmed using EFA |  |  |  |  |  |
| eHEALS  (ID 41) [52] | 2 factors^f^: (*n*=1,215)  1 out of 1 was confirmed using EFA | Cronbach's alpha: (*n*=1,215)  Factor 1: .81,  Factor 2: .73 |  |  |  | 0 out of 1 hypothesis confirmed: (*n*=1,215)  -with age groups |
| eHEALS  (ID 42) [53] | 2 factors^g^: (*n*=188)  0 out of 1 was confirmed using EFA | Cronbach’s alpha: (*n*=188)  Factor 1: .90  Factor 2: .77 |  |  | 0 out of 4 hypotheses were confirmed. (*n*=188)  -with Newest Vital Sign: *r*=.07  -with General Self-Efficacy Scale: *r*=.28  -with Irritational Health Belief Scale: *r*=-.11  -with Need for Cognition Scale: *r*=.11 |  |
| eHEALS  (ID 43-45)  [54-56] | 3 factors^h^: (*n*=3,176)  3 out of 3 were confirmed using CFA | Cronbach's alpha:  Factor 1: .82-.88, Factor 2: .88-.90, Factor 3: .81-.87 (*n*=996) | 1 out of 1 Multi-group CFA was confirmed across countries (*n*=996) | ICC=.61 (*n*=74) | 5 out of 9 hypotheses were confirmed (*n*=2,180).  -with health-promoting behaviors  -with nursing performance quality  -with age  -with usefulness  -with importance  -with the domain 5, and 8 of the Health Literacy Questionnaire  -with mental, physical components of the health status | 1 out of 1 hypothesis was confirmed (*n*=1,695)  -with the use of internet |
| eHEALS  (ID 46)  [57] | 3 factors^i^: (*n*=256)  1 out 1 was confirmed using CFA |  |  |  |  |  |
| eHEALS  (ID 47) [58] | 3 factors^j^:  1 out of 1 was confirmed using CFA (*n*=829) | Cronbach's alpha: (*n*=829)  Information awareness: .84, Information seeking: .88, Information engagement: .84  Omega: (*n*=829)  Information awareness: .84, Information seeking: .88, Information engagement: .84 | 1 out of 1 Multi-group CFA was confirmed across generation groups (*n*=829) |  |  | 1 out of 1 hypothesis was confirmed (*n*=829).  -with age groups. |
| eHEALS  (ID 48) [39] | Bifactor model (general factor with two group factors)^k^: (*n*=703)  1 out of 1 was undetermined. | Omega for a general factor: .99, but no information for the two group factors (*n*=703). |  |  |  |  |
| eHEALS-E  (ID 49) [59] | 6 factors: (*n*=644)  1 out of 1 was confirmed using CFA | Cronbach's alpha:  Factors: .52-.81 (*n*=644) |  |  |  | 0 out of 1 hypothesis was confirmed (*n*=644).  -with user typotology. |
| e-HLS  (ID 50) [60] | 3 factors: (*n*=710)  0 out of 1 was confirmed using CFA |  |  |  | 3 out of 7 hypotheses were confirmed (*n*=710):  -with empowerment: *r*=.40  -with negative effect: *r*= -.12  -with positive health interaction: *r*=.29  -with health communication: *r*=.43  -with nonadherence: *r*=.45  -with health problem: *r*=.13  -with strain health  -with interaction: non-significance |  |
| DHLI  (ID 51) [61] | 7 factors: (*n*=200)  1 out of 1 was confirmed using EFA | Cronbach's alpha: (*n*=200)  7 factors: .57-.89 (6 out of 7 subscales were supported) |  | ICC=0.84-0.88 (*n*=67) | 4 out of 8 hypotheses were confirmed (*n*=200):  -with age: *r*_s_=-.41  -with internet use: *r*_s_=.39  -with health-related internet use: *r*_s_=.27  -with health perception: *r*_s_=.23  -with physical functioning: *r*_s_=.27  -with mental well-being: *r*_s_=.17  -with health literacy: *r*_s_=.31  -with eHealth literacy: *r*_s_=.51 |  |
| DHLI  (ID 52) [42] | Five factors: (*n*=180)  1 out of 1 was confirmed using CFA |  |  | ICC=0.84 (*n*=89) | 1 out of 2 hypotheses were confirmed (*n*=180):  -with attitude towards internet health information: *r*=.39  -with health stratus: *r*=.27 |  |
| eHLA  (ID 53) [62] | 7 factors: (*n*=475)  0 out of 7 IRT were confirmed their unidimensionality of each factor |  |  |  |  |  |
| eHLQ  (ID 54,55) [63] | 7 factors: (*n*=950)  1 out of 2 were confirmed using CFA, and IRT | Cronbach's alpha: (*n*=475)  7 factors: .77-.86 | 2 out of 2 DFI were confirmed by age and gender (but no statistical values) |  |  |  |
| TeHLI  (ID 56,57) [64] | 4 factors: (*n*=586)  2 out of 2 were confirmed using CFA, and Rasch analysis | Cronbach's alpha:  4 factors: .87-.92 (*n*=283)  Omega:  4 factors: .92-.96 (*n*=283)  Item reliability: .81-.91  Item separation index: 4.37-10.6 (*n*=283) |  |  | 20 out of 37 hypotheses were confirmed (*n*=283):  -with task/technology-oriented characteristics  -with AAHLS 3 subscales  -with eHEALS 4 subscales  -with age |  |

ID, study identification number (a study identification number was assigned to each of the 57 studies in the 41 articles because some articles covered multiple studies); IRT, Item response theory.

^a^ information seeking (items 1, 2, 3, 4, 5, 8), information appraisal (items 6, 7) [44,31,39]

^b^ factor 1 (items 1, 2, 4), factor 2 (items 3, 5, 6, 7, 8) [45]

^c^ factor 1 (items 3, 4), factor 2 (items 1, 2, 5, 6, 7, 8) [46]

^d^ factor 1 (items 1, 2, 3, 4, 5), factor 2 (items 6, 7, 8) [47–50]

^e^ factor 1 (items 2, 6, 7, 8), factor 2 (items 1, 3, 4, 5) [51]

^f^ factor 1 (items 1, 2, 3, 4), factor 2 (items 5, 6, 7, 8) [52]

^g^ information acquisition (items 1, 3, 4), information application (items 2, 5, 6, 7, 8) [53]

^h^ awareness (items 3, 4), skills (items 1, 2, 5), evaluation (items 6, 7, 8) [54–56]

^i^ awareness (items 1, 2), skills (items 4, 5), evaluation (items 6, 7, 8) [57]

^j^ information awareness (items 3, 4), information seeking (items 1, 5), information engagement (items 2, 6, 7, 8) [58]

^k^ general factor (items 1, 2, 3, 4, 5, 6, 7, 8), subfactor 1 (items 1, 2, 3, 4, 5, 8), subfactor 2 (items 6, 7) [39]

(The above item numbers are those assigned in the original article by Norman and Skinner [24])
